# CT scan findings impact on hearing thresholds in otosclerosis: A study of 108 patients

**DOI:** 10.1016/j.amsu.2022.103716

**Published:** 2022-05-02

**Authors:** Sara Halily, Bushra Abdulhakeem, Youssef Oukessou, Sami Rouadi, Reda Abada, Mohamed Roubal, Mohamed Mahtar

**Affiliations:** ENT Head and Neck Surgery Department, Ibn Rochd University Hospital, Faculty of Medicine and Pharmacy, Hassan II University, Casablanca, Morocco

**Keywords:** Case series, Otosclerosis, Bone conduction, Air conduction, Temporal bone CT scan

## Abstract

**Background:**

The relationship between the location of otosclerotic zones and hearing thresholds has been evaluated in several studies and has generated different conflicting reports. This study was carried out in order to evaluate the relationship between otosclerotic zones extension on CT scan and pure tone audiometry (PTA) thresholds, before and after stapedotomy. Materials and Methods: 108 patients with a positive surgical diagnosis of otosclerosis, operated by the same surgeon, were enrolled in this retrospective study, performed in a tertiary referral hospital between 2015 and 2018.

**Results:**

PTA thresholds were significantly poorer in cases of extensive otosclerosis (peri cochlear, peri vestibular, or internal auditory canal hypodensities, p = 0,001). However, for cases with hypodensity extending to the endosteum of cochlea (Type III), we have noted a significant improvement in postoperative PTA thresholds (Mean AC (air conduction) = 32,8 ± 8,16/62,97 ± 12,28 dB), Mean BC (bone conduction) = 18,3 ± 8,56/26,25 ± 15,93 dB). Conclusions: In our study, extensive and multifocal otosclerosis lesions had a statistically significant negative impact on postoperative AC and BC threshold; however, type III lesions tend to be associated with a very good prognosis.

## Highlights

• Evaluate the relationship between otosclerotic foci extension on temporal bone CT scan and pure tone audiometry (PTA) thresholds, before and after stapedotomy.
• There are no studies up to date that report a perfect and strict correlation between radiological expression and audiometric thresholds.
• In our series, we have objectified the existence of a negative impact of extensive peri cochlear and/or peri vestibular foci on audiometric thresholds, with a weak tendency to improve and sometimes even to worsen bone thresholds.

## 1 Introduction

Otosclerosis is a hereditary bone dysplasia characterized by primitive osteodystrophy selectively affecting the endochondral bone of the otic capsule. It is more common in the Caucasian and predominantly female population. Although recent studies have demonstrated the importance of genetic predisposition in otosclerosis [1], the involvement of other etiopathogenic factors including hormonal, viral, and autoimmune factors remain highly controversial. Fissula ante fenestrum, a preferred site, is affected in about 90% of cases, resulting in conductive hearing loss due to stapedial fixation. Neurosensory hearing impairment is still not clearly elucidated, and normally manifests itself only when the cochlear endosteum is affected by extensive foci of otosclerosis. However, according to some authors, endosteal damage may impact hearing thresholds even in the case of minimal lesions [2].

The diagnosis of otosclerosis is clinical and audiometric. Temporal bone CT scan is not essential for diagnosis but in most cases confirms hypodensity in the anterior part of the platinum [3]. Other anatomical localizations may coexist, such as peri cochlear and/or peri vestibular involvement.This work aims to analyze the impact of the radiological stages, the location and extension of otosclerotic lesions, on pre- and post-operative audiometric thresholds in patients operated for otosclerosis.

## 2 Materials and methods

This study was reviewed by the ethics committee of the HASSAN II university of Casablanca, faculty of medicine (ethical number: 310/2015AB24). We endorse the principles embodied in the Declaration of Helsinki (2013) and expect that all investigation involving human materials are performed in accordance with these principles. Research registry number: researchregistry7586 https://www.researchregistry.com/browse-the-registry#home/?view_2_page=1&view_2_sort=field_2|desc&view_2_search=7586.

We conducted a retrospective study (case series) carried out in a tertiary center, at the university hospital Ibn Rochd Casablanca Morocco, spread over four years from the January 1, 2015 to the December 31, 2018. All data were collected from patient's medical reports. 108 patients with a positive surgical diagnosis of otosclerosis, ten patients had diabetes, 19 had high blood pressure and 20 patients had medical history of smoking cigarettes. All of them had undergone a stapedotomy by the same senior surgeon with 20 years of experience in this field, specialized in otoneurology, with a previous fellowship in Canada.

The diagnosis of otosclerosis was based on PTA (pure tone audiometry) showing conductive or mixed hearing loss, with normal eardrums and abolished stapedial reflexes, whether the symptoms were unilateral or bilateral.

Clinical, audiometric, radiological, and surgical data were collected for all patients from their medical reports.

PTA was performed in all patients one month before surgery and two months postoperatively.

The audiometric parameters collected were as follows: air (AC) and bone (BC) conduction thresholds for frequencies 500, 1000, 2000, and 4000 Hz and air-bone gap (ABG). The audiometric data were collected and compared to the recommendations of the Committee of Hearing and Equilibrium of the American Academy of Otolaryngology-Head and Neck Surgery (AAO- HNS) [4].

In our institution, temporal bone CT scan is systematically performed in case of any conductive or mixed hearing loss with a normal eardrum. Thus, we obtained axial and coronal sections with a thickness of 0.6 mm in all patients. We adopted the Veillon radiological classification to characterize and evaluate the location and extent of the lesions presented in CT scan. Our study population was divided into four groups. The first group corresponds to patients with no CT-diagnosed otosclerosis or any other middle and inner ear abnormality (Group I: negative imaging), the second group corresponds to patients with minimal lesions without cochlear contact which may correspond to either footplate thickening or pre-stapedial foci without cochlear contact (Group 2: types Ia, Ib, and II in the Veillon classification). The third group corresponds to patients with otosclerosis foci in contact with the cochlear lumen, i.e. with minimal damage to the cochlear endosteum. (Group 3: type III in the Veillon classification), and finally, the fourth group corresponding to patients with multifocal and extensive peri-cochlear and/or peri-vestibular lesions (Group 4: types IVa and IVb in the Veillon classification). We evaluated and compared the audiometric results of the four groups and verified the existence of statistically significant differences in pre-and post-operative ABG, AC, and BC thresholds.

Pre-intervention consideration and patient optimization measures had taken before surgery for all the diabetics and patients with high blood pressure and for others under anticoagulants. All the patients were psychologically assist. Surgery was performed under general anesthesia, with a transcanal approach consisting on a stapedotomy calibrated at 0.6 mm using mainly CO2 laser, or trephine when there was a contraindication to the use of the laser (exuberant extensive foci or significant protrusion of the facial nerve), with the insertion of a 0.4 mm diameter Teflon piston. All the patients were discharged one day after surgery without any complications. The patients were followed-up one-month post operatively they were clinically examined. The second month, they were assessed by having PTA. The results were shown below.

The statistical analysis was performed using SPSS statistics (version 24), using the Annova one-factor test to compare the means between the four groups, as well as the Chi-square test. The statistical significance level was 0.05.

CT scan Veillon classification: [4].

Type 1a: Footplate thickening >0.6 mm only.

Type 1b: Pre-stapedial hypodensity less than or equal to 1 mm.

Type II: Pre-stapedial hypodensity greater than 1 mm, without contact with the cochlear lumen.

Type III: Pre-stapedial hypodensity greater than 1 mm, in contact with the cochlear lumen.

Type IVa: Peri cochlear hypodensities in the middle layer of the labyrinthine capsule.

Type IVb: Posterior labyrinthine hypodensities around the lumen of the semicircular canals or vestibule. This case series has been reported in line with the PROCESS Guideline 2020. [5].

## 3 Results

During the study period, 108 patients were operated on, representing 137 ears. The mean age was 43.31 years (±10.82) with extremes ranging from 23 to 74 years. 67.9% of the patients were female. Ten patients had diabetes, 19 had high blood pressure and 20 patients had medical history of smoking cigarettes. The mean duration of disease progression at the time of surgery was 5.7 years (±4.57) with extremes ranging from one year to 20 years. Otosclerosis was bilateral in 78.6% of patients. Tinnitus was present in 68.5% of patients.

The mean preoperative AC threshold was 61.04 dB (±11.24). The mean preoperative BC threshold was 23.82 dB (±10.4). The mean preoperative ABG was 37.43 dB (±8.66).

Concerning the imaging data, of the 137 CT scans performed, 13 (9.5%) were normal, 7 (5.1%) showed a thickened footplate, 14 (10.2%) showed a pre stapedial focus of less than 1 mm, 65 (47.5%) showed pre stapedial hypodensity >1 mm without cochlear contact, 27 (19. 7%) showed supra-millimetric pre stapedial hypodensity in contact with the cochlear lumen, 9 (6.5%) revealed peri cochlear hypodensities, and finally, hypodensities around the vestibule and semicircular canals were found in 2 patients (1.5%).

Precautionary measures were taken to avoid post-operative complications.

The postoperative audiometric evaluation was performed after 2 months and showed a clear improvement of the mean auditory thresholds in AC and BC (35.22 dB (±13.79) and 17.01 dB (±9.65) respectively). The postoperative mean ABG (air bone gap) was 17.5 dB (±8.62).

**Fig. 1:**
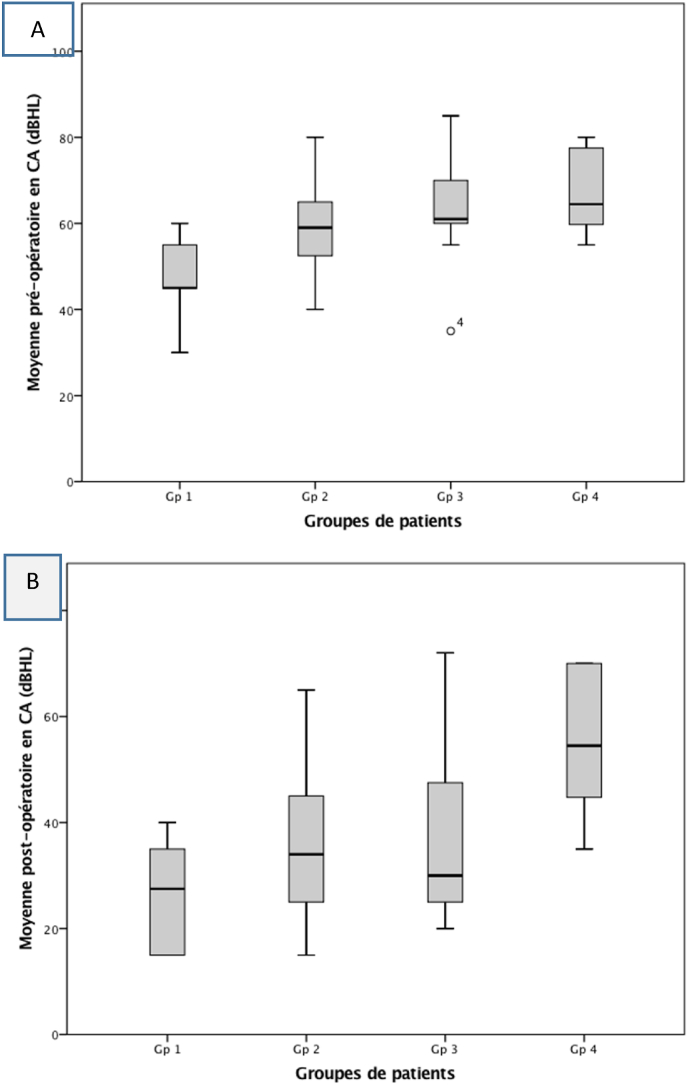
Mean air conduction (AC) according to the four patient groups. A: preoperative. B: postoperative.

**Fig. 2:**
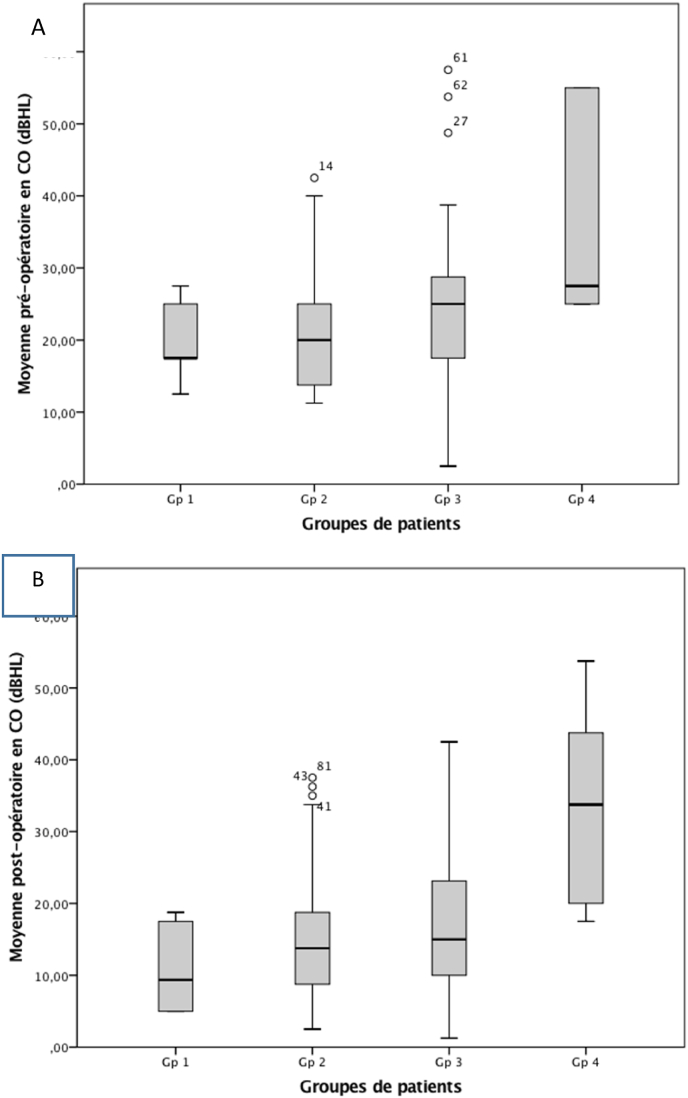
Mean bone conduction (BC) according to the four patient groups. A: preoperative. B: postoperative.

The study of the relationship between the audiometric parameters and the imaging data showed that the more advanced the radiological stage was, the worse the preoperative audiometric threshold. Thus, hearing loss was more pronounced in patients with extensive lesions. These differences were statistically significant for AC (p < 0.001 - ANOVA), and BC (p = 0.004 - ANOVA). The mean preoperative ABG for the four groups were 29.50 dB (±9.84); 36.77 dB (±8.85); 37.09 dB (±7.19) and 39.80 dB (±5.14) respectively. These differences reached statistical significance (p = 0.006- ANOVA) (Table 1) (Fig. 1, Fig. 2).

**Table 1:** Pre and postoperative audiometric data of patients based on imaging results.

Paramètres	Groupe 1	Groupe 2	Groupe 3	Groupe 4	p
Preoperative mean air conduction thresholds (SD)	48.50 dB (9.15)	57.85 dB (9.77)	63.97 dB (11.33)	68.71 dB (10.79)	<0.001
Preoperative mean bone conduction thresholds (SD)	19.75 dB (5.36)	21.3 dB (8.16)	26.05 dB (15.03)	35.83 dB (14.88)	0.004
Preoperative air-bone gap (SD)	29.50 dB (7.09)	36.77 dB (8.38)	37.9 dB (7.19)	39.80 dB (5.14)	0.006
Postoperative mean air conduction thresholds (SD)	25.50 dB (9.84)	33.19 dB (12.7)	35.34 dB (17.23)	52.57 dB (15.67)	<0.001
Postoperative mean bone conduction thresholds (SD))	11.5 dB (8.20)	15.71 dB (8.14)	17.96 dB (11.08)	32.21 dB (15.55)	<0.001
Postoperative air-bone gap (SD)	11.25 dB (6.46)	15.98 dB (8.01)	19.50 dB (7.19)	26.01 dB (7.67)	0.005
Mean ABG closure	17.02 dB (5.37)	20.64 dB (10.76)	19.60 dB (8.62)	12.18 dB (6.1)	0.333

SD: standard deviation.

Concerning the postoperative audiometric results, we also noted that the thresholds were worse in patients with extensive lesions. These differences were statistically significant for AC (p < 0.001 - ANOVA), as well as for BC (p < 0.001 - ANOVA). The mean ABG closure for the four groups was 17.02 dB (±5.37), 20.64 dB (±10.76), 19.60 dB (±8.62), and 12.18 dB (±6.1), respectively. These differences did not reach statistical significance (p = 0.333) (Table 1) (Fig. 1, Fig. 2).

Comparison of the average hearing gain in terms of AC and BC between the four groups for all frequencies showed on the one hand that the averages were almost similar between the first three groups, and on the other hand that they were significantly higher than those of the fourth group. These differences reached statistical significance for the average BC gain at 2000 Hz only (p = 0.008) (Table 2, Table 3).

**Table 2:** Comparison of average AC gain between the four groups.

Average AC gain	Group 1	Group 2	Group 3	Group 4	p
500 Hz (DS)	29.00 dB (12.2)	27.27 dB (14.78)	30.79 dB (13.66)	15.00 dB (7.63)	0.081
1000 Hz (DS)	26.00 dB (8.76)	25.98 dB (13.5)	24.95 dB (14.37)	15.32 dB (7.24)	0.201
2000 Hz (DS)	20.00 dB (10.02)	21.14 dB (13.23)	22.37 dB (9.33)	10.00 dB (8.2)	0.121
4000 Hz (DS)	17.45 dB (8.64)	21.74 dB (13.9)	21.84 dB (9.45)	7.14 dB (2.67)	0.061

SD: Standard deviation.

**Table 3:** Comparison of average BC gain among the four groups.

Average BC gain	Group 1	Group 2	Group 3	Group 4	p
500 Hz (DS)	8.50 dB (6.68)	5.24 dB (7.50)	6.84 dB (9.01)	4.12 dB (5.6)	0.200
1000 Hz (DS)	8.52 dB (5.1)	6.2 dB (7.8)	7.63 dB (10.45)	4.8 dB (4.49)	0.400
2000 Hz (DS)	12.50 (6.52)	9.24 dB (11,27)	9.42 dB (10.67)	−2 dB (4.32)	0,008
4000 Hz (DS)	5.50 dB (7.97)	4.39 dB (8.34)	9.47 dB (12.8)	1.2 dB (3.2)	0,050

SD: Standard deviation.

As for the sensorineural component of hearing loss, we analyzed the postoperative BC thresholds to identify the existing differences between the four groups. None of the patients in our series worsened their BC threshold by more than 10 dB. In the group of patients with normal CT scans, no patient worsened his BC threshold, 46.1% of the cases had a gain greater than 10 dB. In the group of patients with minimal lesions, one (4.7%) worsened his BC threshold by more than 5 dB, 16.4% of the cases had a gain greater than 10 dB. In the group of patients with cochlear lesions, only one patient (3.7%) worsened his BC threshold by more than 5 dB, with 52.6% of the cases having a gain greater than 10 dB. In the group of patients with extensive lesions, the BC threshold decreased by more than 5 dB in 27.2% of the cases. The gain did not exceed 5 dB in any of these patients. Therefore, the risk of worsening BC thresholds tends to be higher in patients with extensive lesions (p = 0.001, Pearson Chi-square).

In terms of postoperative mean BC thresholds, we found that the poorest audiometric results were found in patients in the fourth group mainly for conversational frequencies. For the 1000 Hz frequency, the mean postoperative BC thresholds for the four groups were 11.00 dB (±6.14), 15.23 dB (±8.53), 15.79 dB (±11.21), and 30.71 dB (±13.67) respectively. These differences were statistically significant (p < 0.001 -ANOVA). For the 2000 Hz frequency, the mean postoperative BC thresholds for the four groups were 12.00 dB (±8.56), 18.41 dB (±11.27), 22.63 dB (±12.73), and 44.29 dB (±16.69) respectively. These differences reached a statistically significant level of significance (p < 0.001 -ANOVA).

## 4 Discussion

Otosclerosis is a primitive osteodystrophy of the bone labyrinth selectively affecting the endochondral bone of the otic capsule. Although its diagnosis is mainly based on anamnestic, clinical, and audiometric arguments, temporal bone CT scan remains fundamental. On the one hand, it confirms the diagnosis by visualizing the otosclerotic site and on the other hand, it eliminates possible differential diagnoses such as House syndrome, ossicular chain lysis, congenital cholesteatoma, Gusher syndrome, or facial neurinoma that could block the stapes [3].

The determination of the sensitivity and specificity of temporal CT scan in the diagnosis of otosclerosis has been the subject of several studies. The results were not always unanimous among the authors. Dudau et al., in a recent study, reported that the specifity of temporal bone CT scan in detecting otosclerosis foci was possible in 63% of cases, resulting in a diagnosis for every 1.6 scans performed. In terms of therapeutic impact, the authors found that the CT scan diagnosis resulted in surgery in 24% of cases, which is equivalent to one intervention for every 4 scans performed [6]. Finally, in a recent literature review [7], the authors report that the sensitivity of temporal bone CT scan in detecting otosclerosis foci varies considerably, ranging from 34% to 95%, and remains very limited in foci with a diameter not exceeding 1 mm, superficial foci, in inactive otosclerosis and when the variations in density do not exceed 200 Hounsfield Units (HUs) [[8], [9], [10], [11]]. The authors also report that it has been demonstrated (Evidence Level III and IV according to the Oxford center of Evidence-based Medicine guidance) that quantitative measures of bone density around the otic capsule were lower in patients with otosclerosis than in controls, which may suggest that temporal bone CT scan would allow a semi-automatic diagnosis of otosclerotic lesions [[12], [13], [14]].

Numerous radiological classifications have been proposed over the years, but none of them is universally accepted and adopted. In our series, we have used the Veillon radiological classification, which makes it possible both to locate lesions and to assess their extent. For a classification to be relevant, it is, therefore, necessary that it can help clinical management of the disease, predict prognosis and surgical outcome [7].

The correlation between radiological stages and pre-and post-operative audiometric thresholds in otosclerosis remains controversial due to the different published results. Despite the limitations of the studies, many of them report that extensive otosclerotic foci that are not limited to the oval window on temporal bone CT have a significant negative impact on preoperative audiometric thresholds which turn out to be worse when compared to healthy subjects or patients with otosclerosis with normal imaging [[15], [16], [17]]. BC audiometric thresholds would therefore be worse when the focus reaches the peri-cochlear region, cochlear endosteum, vestibule, round window, or internal auditory canal. Other studies refute the possibility of a correlation between imaging and audiometric data, mainly between the sensorineural component of hearing loss and cochlear involvement, but rather support the association between BC thresholds and the size of otosclerotic foci, when the latter is limited to the oval window [10,18].

In our series, we found that preoperative audiometric thresholds in AC and BC tend to increase as the radiological stage progresses, with the worst thresholds found in patients with cochlear lumen contact lesions and those with peri cochlear and peri vestibular lesions. Concerning postoperative thresholds, we similarly noted an increase in thresholds in the advanced stages, with the worst results obtained in patients with extensive peri cochlear and/or peri vestibular lesions, in whom the mean threshold in AC was 68.71 dB (±10.79) dB and in BC 35.83 (±14.88) dB. These differences were highly significant (p = 0.002 -CA, p < 0.001 - CO). The analysis of postoperative bone thresholds allowed us to conclude that patients with extensive lesions were the least able to improve their threshold and were at the greatest risk of worsening it since none of the patients in this group had a BC hearing gain over 5 dB and 27.2% had worsened their threshold by more than 5 dB.

Regarding the third group, although they had higher preoperative hearing thresholds in AC and BC compared to the first two groups, we found that postoperatively these patients were able to improve their bone thresholds significantly as 52.6% of them had a BC gain of more than 10 dB, and only one patient (4.7%) had worsened his bone threshold by more than 5 db. This finding was confirmed when we compared postoperative hearing thresholds and average hearing gains in terms of AC and BC between the four groups for all the studied frequencies. We, therefore, objectified that patients with minimal cochlear endosteal lesions had almost similar postoperative thresholds and hearing gains in terms of AC and BC to patients in the first two groups, in some cases even better. All these findings led us to conclude that otosclerosis surgery in patients with cochlear lumen contact lesions, i.e. when there is minimal damage to the cochlear endosteum, was correlated with a good functional prognosis, in contrast to patients with extensive peri cochlear and/or peri vestibular lesions in whom audiometric results are not satisfactory. These results emphasized the impact of the Veillon classification. Marx et al. [15], in a study analyzing the correlation between imaging results and audiometric thresholds in otosclerosis, reported similar results to ours by revealing that postoperative audiometric thresholds in AC and BC were worse in patients with extensive and multifocal lesions and that these patients had a low chance of improvement and a higher risk of worsening their bone threshold compared to patients with minimal pre-stapedial localized lesions or those with normal CT scans. The relationship between the location and extent of otosclerotic foci and audiometric thresholds has also been the subject of several histopathological studies. While Schuknecht and Barber [19] found almost no correlation between cochlear involvement and bone audiometric thresholds, Hueb et al. [2], in a series of 37 temporal bones, showed a positive correlation between size, lesion activity, cochlear endosteal involvement, and bone thresholds. Conclusion: There are no studies up to date that report a perfect and strict correlation between radiological expression and audiometric thresholds, however, many authors agree that extensive multifocal otosclerotic foci provide a poor auditory prognosis.

The correlation between radiological stages and pre-and post-operative audiometric thresholds in otosclerosis remains controversial due to the different published results. There are no studies up to date that report a perfect and strict correlation between radiological expression and audiometric thresholds this why we have conducted this current study and we have objectified the existence of a negative impact of extensive peri cochlear and/or peri vestibular foci on audiometric thresholds, with a weak tendency to improve and sometimes even to worsen bone thresholds. We also found that in the group of patients with minimal lesions of the cochlear endosteum, the postoperative results were almost similar to those with minimal and localized pre-stapedial lesions and those with normal imaging, which confers a good functional prognosis to this group. However, we know for sure that Further studies should be carried out to confirm this relationship by collecting a large number of patients why not having a large metaanalysis and systematic review.

## 5 Conclusions

In our study, extensive and multifocal otosclerosis lesions had a statistically significant negative impact on postoperative AC and BC threshold; however, type III lesions tend to be associated with a very good prognosis.

## Sources of funding

None.

## Ethical approval

This study was reviewed by the ethics committee of the HASSAN II university of Casablanca, faculty of medicine. We endorse the principles embodied in the Declaration of Helsinki (2013) and expect that all investigation involving human materials are performed in accordance with these principles.

## Consent

Written informed consent was obtained from patients for publication. Copies of the written consent are available for review by the Editor-in-Chief of this journal on request.

## Author contribution

Halily sara, bushra abdulhakeem: study concept data collection, writing the paper, and making the revision of the paper following the reviewers instruction.

Youssef oukessou, sami rouadi, reda abada, mohamed roubal, mohamed mahtar: reviewing and validating the manuscript credibility.

## Registration of research studies

Name of the registry: research registry
Unique Identifying number or registration ID: researchregistry7586
Hyperlink to your specific registration (must be publicly accessible and will be checked): researchregistry7586https://www.researchregistry.com/browse-the-registry#home/?view_2_page=1&view_2_sort=field_2|desc&view_2_search=7586

## Provenance and peer review

Not commissioned, externally peer-reviewed.

## Guarantor

Halily sara.

## Declaration of competing interest

None.
